# Establishment of MOS-SF36 percentile ranks in the general youth French population

**DOI:** 10.1186/s40359-022-00786-9

**Published:** 2022-03-20

**Authors:** Arthur Trognon, Emilie Tinti, Blandine Beaupain, Jean Donadieu, Michel Musiol

**Affiliations:** 1 Clinicog, Nancy, France; 2 CNRS, ATILF, Lorraine University, Nancy, France; 3 Lorraine University, Nancy, France; 4 French Reference Center for Langerhans Cell Histiocytosis, AP-HP, Trousseau Hospital, Paris, France; 5 INRIA, Nancy-Grand Est, France

**Keywords:** French population, Mental health scale, Physical health scale, Quality of Life

## Abstract

**Background:**

The SF-36 is a generic quality of life questionnaire, massively translated and widely used to obtain physical and mental health status. However, validation work in the French language was carried out over a generation ago. The objective of this study was to obtain the norms of the SF-36 in the French young population.

**Method:**

The sample consisted of 958 non-pre-screened French people aged between 18 and 24 years.

**Results:**

The internal consistencies of the scales were high and the metrics associated with the factor structure were satisfactory. In general, women presented significantly higher scores than men.

**Conclusion:**

Our results suggest that the SF-36 remains a reliable tool for studying quality of life in the young French population.

**Supplementary Information:**

The online version contains supplementary material available at 10.1186/s40359-022-00786-9.

## Introduction

Since the 1990s, health-related quality of life has gradually become a major theme in clinical research [1]. Indeed, although the health status of a population is most often expressed in quantitative terms such as life expectancy, mortality, or morbidity, a growing number of studies are now interested in measuring health status, and in particular its relationship with quality of life. Nowadays, the patient's perceived quality of life is placed at the center of the care process. It can thus reflect the satisfaction and perceived benefits of an intervention, which could not necessarily be measured by other parameters [2]. Thus, the importance of quality of life assessment is such that it has led to the establishment of indicators centered on patient-reported outcomes measures (PROMs) by the French High Authority for Health (HAS) [3]. These indicators are beginning to be used by regulatory and reimbursement authorities, who require them as part of the decision-making process [4].

Thus, a large number of self-reported questionnaires have been developed to measure these dimensions, notably the very broad Medical Outcomes Questionnaire 149-item from the RAND Health Insurance Experiment [5].

Several tools have been developed from this original questionnaire, including the derived SF-20 and SF-36 versions, which have shown more precise discriminatory abilities in their validation studies [6]. Thus, the Medical Outcomes Study Short Form (SF-36) has become one of the most widely used self-reported quality of life questionnaires for assessing health status, given its discriminatory properties of well-being at the level of clinical groups [7], and has thus been used extensively in the monitoring of clinical practice outcomes and medical treatment effects. This questionnaire measures quality of life on the basis of eight dimensions or concepts that are frequently used in health studies. These eight dimensions are estimated from eight subscales that examine general health; mental health (with respect to anxiety and depression components); physical functioning; limitation of work capacity or daily activities due to physical functioning as well as that due to emotional disorders; vitality; pain; and social functioning. The SF36 has already been evaluated numerous times for its differential performance in comparison with other perceived quality of life questionnaires in different clinical settings, including the Euroqol questionnaire [8]; the Sickness Impact Profile [9]; and the Hopkins Symptom Checklist 25 [10] with similar qualities. However, it would appear that the SF-36 stands out for its operational qualities in the assessment of general health, as well as its ease and speed of administration [9, 10].

The questionnaire has already been translated several times into French, and norms have been obtained for the French [11] and Swiss [12] populations, but they were established almost a generation ago. In addition, no work has been done to our knowledge to establish SF-36 norms expressed as percentile ranks. The purpose of this study was to establish the norms of the SF-36 in the youth French population (15–24 years) as percentile ranks and to reassess its psychometric properties in terms of reliability and validity, in order to provide a baseline in the general young population and to provide a tool that can be used in clinical routine.

## Material and methods

### Study design

The questionnaire was adapted in the formulation of the items from the version proposed by (Richard et al. 2000) [12]. It was then computerized using the Google Form tool. Sampling was carried out randomly by distributing the questionnaire on social networks, without direct contact with the participants and on the basis of anonymous voluntary contributions. Only age, gender, and date of completion were collected, ensuring complete anonymity for participants. An exclusion criterion in terms of age (> 24 years) was applied after data collection during data preprocessing.

### Subjects

Nine hundred and fifty-eight (n = 958) not preselected adults (mean age = 22.1 years; SD = 1.76) from the general French population participated in this study. Participants screening was completed online, and ethical consents were obtained online in agreement with the Declaration of Helsinki. The study was approved by the “Comité de Protection des Personnes Sud-Est VI”. Full measures were available for all subjects. No minors were included in the study.

### Questionnaire: the MOS-SF36

The SF-36 is a short 36-item behavioural questionnaire measuring eight quality of life dimensions: general health (GH-5 items), vitality (VT-4 items), bodily pain (BP-2 items), limitation of physical problems (RP-4 items), limitation of emotional problems (RE-3 items), mental health (MH-5 items), and physical functioning (PF-10 items), social functioning (SF-2 items). The SF-36 also includes an item to estimate the change in the subject's health status during the year preceding the assessment (HC).

### Scoring

For each dimension, item responses were re-encoded on a scale ranging from 0 (best) to 100 (worst), following the standard SF-36 scoring algorithm [13], adapted for a 5-point Likert scale. The algorithm used is available in Table 1 and the full questionnaire used in the study is available in Additional file 1.

**Table 1:** Scoring algorithm for the SF-36 questionnaire

Item number	Participant’s response	Scoring
1, 2, 20, 22, 34, 36	1	100
	2	75
	3	50
	4	25
	5	0
3, 4, 5, 6, 7, 8, 9, 10, 11, 12	1	0
	2	50
	3	100
13, 14, 15, 16, 17, 18, 19	1	0
	2	100
21, 23, 26, 27, 30	1	100
	2	75
	3	50
	4	25
	5	0
24, 25, 28, 29, 31	1	0
	2	25
	3	50
	4	75
	5	100
32, 33, 35	1	0
	2	25
	3	50
	4	75
	5	100

For the calculation of the composite scores, we averaged the PF, RP, BP and GH subscales for the physical composite score (PCS) and averaged the VT, SF, RE and MH subscales for the mental composite score (MCS).

### Internal consistency and reliability

Internal consistency and reliability of the items were examined by Cronbach’s alpha. Reasonable acceptability criterion was set to .70 ≤ ɑ ≤ .90 with exceeding lower bound meaning a low reliability, and exceeding higher bound meaning too many similar items, decreasing the scale’s true reliability [14, 15].

### Factor structure

In order to test our 8-factors model for SF-36 and assess construct validity, we conducted a confirmatory factor analysis. Generalized least squares method was performed in order to test the fit capability of the factor structure. Model fit was assessed using the following fit indices: we used the χ^2^ test statistic for absolute fit; the comparative fit index (CFI) and Tucker-Lewis Index (TLI) for fit relative to a null model [16–18]; the Standardized Root Mean Square Residual [19] and the Root Mean Square Error of Approximation [20] for overall fit. Accordingly to Hu and Bentler (1999) [17], we assumed that our 8-factors model fit well if CFI > .95; TLI > .95; RMSEA < .06 and SRMR < .08. All statistical analyses were coded in R with Lavaan library and interpreted in RStudio v1.0.143.

## Results

### Descriptive statistics

Descriptive statistics of the study sample are shown in Table 2. Results showed that women reported poorer health compared to men for all variables except for BP.

**Table 2:** Descriptive statistics of the study sample

Parameter	Men (n = 397)	Women (n = 561)
Age	21.43 (± 1.81)	20.87 (± 1.69)
Socio-cultural level	3.74 (± 1.12)	3.6 (± 1.15)
GH	28.1 (± 18.49)	34.59 (± 21.88)
VT	46.82 (± 18.14)	56.99 (± 18.41)
BP	84.73 (± 15.57)	79.05 (± 17.41)
RP	16.69 (± 26.35)	23.8 (± 33.9)
RE	29.89 (± 37.85)	46.76 (± 41.23)
MH	35.09 (± 21.06)	47.17 (± 21.62)
PF	3.36 (± 8.97)	5.76 (± 9.61)
SF	52.7 (± 8.91)	53.34 (± 9.53)
HC	45.97 (± 23.62)	47.5 (± 25.58)
PCS	33.96 (± 9.37)	35.8 (± 11.17)
MCS	42.45 (± 17.21)	51.03 (± 17.63)

### Internal consistency and reliability

Results concerning internal consistency and reliability are presented in Table 3 and Additional file 3: Table S12. Data showed that the SF-36 questionnaire carries high internal consistency and reliability even when an item is dropped.

**Table 3:** Internal consistency and reliability for the SF-36 questionnaire

Subscale	Cronbach’s alpha	Lower confidence bound	Upper confidence bound
GH (5 items)	.78	.75	.79
VT (4 items)	.81	.79	.83
BP (2 items)	.80	.77	.82
RP (4 items)	.78	.76	.80
RE (3 items)	.80	.78	.82
MH (5 items)	.87	.86	.88
PF (10 items)	.84	.79	.82
SF (2 items)	.85	.83	.86

The Cronbach alpha was measured at .88 [CI_95%_ = .87–.89] for the full SF-36 questionnaire. When each of the SF-36 items was removed from the analysis in order to assess robustness, Cronbach’s alpha remained high (varying from .87 to .89 with mean_ɑ_ = .88, SD = .007; Additional file 3: Table S12). Measures for the subscales ranged from .78 to .85. All measures were above the minimum acceptable rate of .70 and was close to the maximum expected value of .9 (Table 3).

### Confirmatory factor analysis

Confirmatory factor analysis suggested that the 8-factor model fit well with the SF-36 questionnaire, except for the CFI and TLI which remains slightly below the pre-defined cut-off [*χ*^2^_(595)_ = 2247, *p* < .001, CFI = .89, TLI = .88, RMSEA = .058, SRMR = .053]. We assumed that, based on these indices, this sample has an acceptable fit to the 8-factor model.

Additional file 3: Table S13 shows the standardized factor loadings for the SF-36. The analysis revealed factor loadings in the range of .5 to .86 for the GH factor, .69 to .85 for the MH factor, .69 to .73 for the VT factor, .77 to .87 for the BP factor, .85 to .87 for the SF factor, .69 to .81 for the RE factor, .62 to .73 for the RP factor, and .41 to .68 for PF.

### Justification of the normative approach

A two-ways ANOVA (dimension * gender) on the measured score showed a significant effect of gender (F_(1,41202)_ = 84.75, *p* < .001), dimension (F_(8,41202)_ = 8942.02, *p* < .001), and a significant interaction between gender and dimension (F_(8,41202)_ = 18.59, *p* < .001). Since this significant interaction indicated that the distribution within the dimensions of the SF-36 was directly dependent on the factor of gender, we decided to separate them in the setting of the norms.

### Normative values

Normative data for the SF-36 composite scores expressed in percentiles are presented in Table 4. The full percentiles for the 8 subscales, the 2 composite factors and HC item are available in Additional file 3: Tables S1–S11. Women showed higher scores compared to men for each scale except for BP.

**Table 4:** Normative data for the SF-36 composite scores expressed in centiles

SF-36 scale percentiles	Men	Women
Mental composite score (MCS)		
25	28.12	36.46
50	37.18	50.41
75	51.98	65.31
99	78.45	83.875
Physical composite score (PCS)		
25	26.875	27.5
50	31.25	32.5
75	37.5	41.875
99	58.85	66.875

## Discussion

The present study verified the reliability and the internal consistency of the French version of the 36-Item Short Form Survey (SF-36) questionnaire in a young population.

Cronbach’s alpha measures suggested that the SF-36 questionnaire was internally reliable, with measured alphas remaining in the .70 ≤ ɑ ≤ .90 interval recommended by Bland and Altman (1997) and DeVellis (2003).

Further confirmatory factor analysis supported the eight-factor structure of the SF-36 questionnaire, with items 1; 33; 34; 35; 36 grouped in the “General Health” factor, items 23; 27; 29; 31 grouped in the “Vitality” factor, items 21; 22 grouped in the “Body Pain” factor, items 13; 14; 15; 16 grouped in the “Role limitation: Physical” factor, items 17; 18; 19 grouped in the “Role limitation: Emotional” factor, items 24; 25; 26; 28; 30 grouped in the “Mental Health” factor, items 3; 4; 5; 6; 7; 8; 9; 10; 11; 12 grouped in the “Physical Functioning” factor, and items 20; 32 grouped in the “Social Functioning” factor. Analysis suggested that this model is close to the standards defined by Hu and Bentler (1999), with only CFI and TLI which remains slightly below the cut-off.

We then performed an analysis of variance that suggested some gender differences in self-reported responses, with women reporting lower quality of life than men for all domains studied except BP. In a general manner, authors commonly agrees that women report a lower quality of life than men [21–23], especially in Western countries where lower quality of life scores were measured in women, in correlation with higher depression and sleep disorder score measures [24]. However, our work is, to our knowledge, the only one to report gender differences between all scales, except for body pain. This observation could be explained by the existing difference between men and women regarding pain perception. Previous studies have indeed shown gender differences regarding the experience of pain [25]. However, it is commonly accepted that women typically report more severe and frequent complaints about pain [26], including in pain thresholding experiments [27], suggesting that women should report higher scores. This lack of significant difference in the Body Pain dimensions could thus be explained by the phenomenon of habituation, measured in experimental pain paradigms [28–30], which would lead women to score more positively on items measuring perceived pain, despite experiencing greater and more frequent pain events overall. This hypothesis is strengthened by the experimental pain literature, some of whose results suggest a more rapid adaptation and habituation to pain in women in contrast to men [31–33], whose effects are objectivable at the neurophysiological level [34].

Finally, we established normative and percentile data for all eight subscales of the SF-36, as well as for its single-item subscale and in its physical and mental composite scores.

## Conclusion

The present work strengthened existing SF36 data regarding its internal consistency in measuring physical and mental health. The study provides norms expressed in percentile ranks for the young French population.

## Study limitations

The study has some limitations. First of all, the representativeness of the sample seems limited, as the observations were collected on the basis of volunteers frequenting the social networks. Moreover, given the lack of contact between the participants and the experimenters, it was not possible to control whether some participants completed the questionnaire more than once, nor estimate the real response rate or evaluate the test–retest reliability. Furthermore, we did not conduct an examination in terms of convergent and discriminant validities. Finally, although the description of the questionnaire clearly identified the target population as the general healthy population, it was not possible to control for the presence of individuals with medical conditions in the sample.

## Supplementary Information

**Additional file 1:** MOS-SF36 scale version used in the present study with associated factors and scaling.

**Additional file 2:** Dataset used in the present study.

**Additional file 3:** Internal consistency and reliability of the MOS-SF36 if an item is dropped.

## Data Availability

The dataset analyzed during the current study is available in Additional file 2 (“sf36_supplementaryFile2.xlsx”).
