# Supplementary material for: Establishment of MOS-SF36 percentile ranks in the general youth French population
Source: BMC Psychol. 2022 Mar 20;10:74. doi: 10.1186/s40359-022-00786-9 (PMC8934506; doi:10.1186/s40359-022-00786-9)
Supplement: Supplementary file 3 — Additional file 3: Internal consistency and reliability of the MOS-SF36 if an item is dropped. [file 40359_2022_786_MOESM3_ESM.docx]

**SUPPLEMENTARY TABLES**

| Centile (GH) | Men | Women |
| --- | --- | --- |
| 1 | 0 | 0 |
| 5 | 0 | 5 |
| 10 | 5 | 10 |
| 15 | 10 | 10 |
| 20 | 10 | 15 |
| 25 | 15 | 20 |
| 30 | 15 | 20 |
| 35 | 20 | 25 |
| 40 | 22 | 25 |
| 45 | 25 | 30 |
| 50 | 25 | 30 |
| 55 | 30 | 35 |
| 60 | 30 | 35 |
| 65 | 35 | 40 |
| 70 | 35 | 45 |
| 75 | 40 | 50 |
| 80 | 40 | 55 |
| 85 | 45 | 60 |
| 90 | 55 | 65 |
| 95 | 60 | 75 |
| 99 | 80 | 90 |

Supplementary table 1 : Normative data for the SF-36 GH subscale expressed in centiles.

| Centile (VT) | Men | Women |
| --- | --- | --- |
| 1 | 6.25 | 18.75 |
| 5 | 18.75 | 25 |
| 10 | 25 | 31.25 |
| 15 | 31.25 | 37.50 |
| 20 | 31.25 | 37.50 |
| 25 | 37.50 | 43.75 |
| 30 | 37.50 | 43.75 |
| 35 | 43.75 | 50 |
| 40 | 43.75 | 50 |
| 45 | 43.75 | 56.25 |
| 50 | 50 | 56.25 |
| 55 | 50 | 62.50 |
| 60 | 50 | 62.50 |
| 65 | 56.25 | 62.50 |
| 70 | 56.25 | 68.75 |
| 75 | 56.25 | 68.75 |
| 80 | 62.50 | 75 |
| 85 | 68.75 | 75 |
| 90 | 68.75 | 81.25 |
| 95 | 81.25 | 87.50 |
| 99 | 87.50 | 98.25 |

Supplementary table 2 : Normative data for the SF-36 VT subscale expressed in centiles.

| Centile (BP) | Men | Women |
| --- | --- | --- |
| 1 | 37.5 | 37.5 |
| 5 | 50 | 50 |
| 10 | 62.5 | 50 |
| 15 | 75 | 62.5 |
| 20 | 75 | 62.5 |
| 25 | 75 | 75 |
| 30 | 75 | 75 |
| 35 | 87.5 | 75 |
| 40 | 87.5 | 75 |
| 45 | 87.5 | 75 |
| 50 | 87.5 | 75 |
| 55 | 87.5 | 87.5 |
| 60 | 87.5 | 87.5 |
| 65 | 87.5 | 87.5 |
| 70 | 100 | 87.5 |
| 75 | 100 | 87.5 |
| 80 | 100 | 100 |
| 85 | 100 | 100 |
| 90 | 100 | 100 |
| 95 | 100 | 100 |
| 99 | 100 | 100 |

Supplementary table 3 : Normative data for the SF-36 BP subscale expressed in centiles.

| Centile (RP) | Men | Women |
| --- | --- | --- |
| 1 | 0 | 0 |
| 5 | 0 | 0 |
| 10 | 0 | 0 |
| 15 | 0 | 0 |
| 20 | 0 | 0 |
| 25 | 0 | 0 |
| 30 | 0 | 0 |
| 35 | 0 | 0 |
| 40 | 0 | 0 |
| 45 | 0 | 0 |
| 50 | 0 | 0 |
| 55 | 0 | 0 |
| 60 | 0 | 25 |
| 65 | 25 | 25 |
| 70 | 25 | 25 |
| 75 | 25 | 50 |
| 80 | 50 | 50 |
| 85 | 50 | 75 |
| 90 | 50 | 75 |
| 95 | 75 | 100 |
| 99 | 100 | 100 |

Supplementary table 4 : Normative data for the SF-36 RP subscale expressed in centiles.

| Centile (RE) | Men | Women |
| --- | --- | --- |
| 1 | 0 | 0 |
| 5 | 0 | 0 |
| 10 | 0 | 0 |
| 15 | 0 | 0 |
| 20 | 0 | 0 |
| 25 | 0 | 0 |
| 30 | 0 | 0 |
| 35 | 0 | 0 |
| 40 | 0 | 33.3 |
| 45 | 0 | 33.3 |
| 50 | 0 | 33.3 |
| 55 | 0 | 66.6 |
| 60 | 33.3 | 66.6 |
| 65 | 33.3 | 66.6 |
| 70 | 66.6 | 66.6 |
| 75 | 66.6 | 100 |
| 80 | 66.6 | 100 |
| 85 | 66.6 | 100 |
| 90 | 100 | 100 |
| 95 | 100 | 100 |
| 99 | 100 | 100 |

Supplementary table 5 : Normative data for the SF-36 RE subscale expressed in centiles.

| Centile (MH) | Men | Women |
| --- | --- | --- |
| 1 | 0 | 8 |
| 5 | 9 | 15 |
| 10 | 10 | 20 |
| 15 | 15 | 20 |
| 20 | 15 | 25 |
| 25 | 20 | 30 |
| 30 | 20 | 35 |
| 35 | 25 | 35 |
| 40 | 25 | 40 |
| 45 | 25 | 45 |
| 50 | 30 | 45 |
| 55 | 35 | 50 |
| 60 | 35 | 55 |
| 65 | 40 | 55 |
| 70 | 45 | 60 |
| 75 | 50 | 65 |
| 80 | 50 | 70 |
| 85 | 60 | 70 |
| 90 | 65 | 75 |
| 95 | 75 | 85 |
| 99 | 90 | 90 |

Supplementary table 6 : Normative data for the SF-36 MH subscale expressed in centiles.

| Centile (PF) | Men | Women |
| --- | --- | --- |
| 1 | 0 | 0 |
| 5 | 0 | 0 |
| 10 | 0 | 0 |
| 15 | 0 | 0 |
| 20 | 0 | 0 |
| 25 | 0 | 0 |
| 30 | 0 | 0 |
| 35 | 0 | 0 |
| 40 | 0 | 0 |
| 45 | 0 | 0 |
| 50 | 0 | 0 |
| 55 | 0 | 5 |
| 60 | 0 | 5 |
| 65 | 0 | 5 |
| 70 | 1 | 5 |
| 75 | 5 | 10 |
| 80 | 5 | 10 |
| 85 | 5 | 10 |
| 90 | 10 | 15 |
| 95 | 15 | 25 |
| 99 | 50 | 42 |

Supplementary table 7 : Normative data for the SF-36 PF subscale expressed in centiles.

| Centile (SF) | Men | Women |
| --- | --- | --- |
| 1 | 37.5 | 25 |
| 5 | 37.5 | 37.5 |
| 10 | 37.5 | 37.5 |
| 15 | 50 | 50 |
| 20 | 50 | 50 |
| 25 | 50 | 50 |
| 30 | 50 | 50 |
| 35 | 50 | 50 |
| 40 | 50 | 50 |
| 45 | 50 | 50 |
| 50 | 50 | 50 |
| 55 | 50 | 50 |
| 60 | 50 | 50 |
| 65 | 50 | 50 |
| 70 | 50 | 62.5 |
| 75 | 62.5 | 62.5 |
| 80 | 62.5 | 62.5 |
| 85 | 62.5 | 62.5 |
| 90 | 62.5 | 62.5 |
| 95 | 62.5 | 62.5 |
| 99 | 75 | 75 |

Supplementary table 8 : Normative data for the SF-36 SF subscale expressed in centiles.

| Centile (MCS) | Men | Women |
| --- | --- | --- |
| 1 | 15.31 | 19.875 |
| 5 | 19.94 | 25 |
| 10 | 22.81 | 28.125 |
| 15 | 24.81 | 30.312 |
| 20 | 26.06 | 32.5 |
| 25 | 28.12 | 36.46 |
| 30 | 29.31 | 38.75 |
| 35 | 30.81 | 41.875 |
| 40 | 32.58 | 44.27 |
| 45 | 34.89 | 46.46 |
| 50 | 37.18 | 50.41 |
| 55 | 40.16 | 53.85 |
| 60 | 44.77 | 57.91 |
| 65 | 45.25 | 59.9 |
| 70 | 48.92 | 62.60 |
| 75 | 51.98 | 65.31 |
| 80 | 56.19 | 68.22 |
| 85 | 62.48 | 71.25 |
| 90 | 67.73 | 75.1 |
| 95 | 72.78 | 79.69 |
| 99 | 78.45 | 83.875 |

Supplementary table 9 : Normative data for the SF-36 MCS composite score expressed in centiles.

| Centile (PCS) | Men | Women |
| --- | --- | --- |
| 1 | 18.725 | 18.75 |
| 5 | 21.875 | 22.5 |
| 10 | 23.75 | 24.375 |
| 15 | 25 | 25.625 |
| 20 | 26.25 | 26.875 |
| 25 | 26.875 | 27.5 |
| 30 | 28.125 | 28.75 |
| 35 | 28.75 | 29.375 |
| 40 | 29.375 | 30 |
| 45 | 30.625 | 31.25 |
| 50 | 31.25 | 32.5 |
| 55 | 32.5 | 33.75 |
| 60 | 33.5 | 35.625 |
| 65 | 34.375 | 37.5 |
| 70 | 35.625 | 39.375 |
| 75 | 37.5 | 41.875 |
| 80 | 39.875 | 45 |
| 85 | 41.625 | 48.75 |
| 90 | 45 | 52.5 |
| 95 | 51.25 | 58.75 |
| 99 | 58.85 | 66.875 |

Supplementary table 10 : Normative data for the SF-36 PCS composite score expressed in centiles.

| Centile (HC) | Men | Women |
| --- | --- | --- |
| 1 | 0 | 0 |
| 5 | 0 | 0 |
| 10 | 0 | 0 |
| 15 | 25 | 25 |
| 20 | 25 | 25 |
| 25 | 25 | 25 |
| 30 | 50 | 25 |
| 35 | 50 | 50 |
| 40 | 50 | 50 |
| 45 | 50 | 50 |
| 50 | 50 | 50 |
| 55 | 50 | 50 |
| 60 | 50 | 50 |
| 65 | 50 | 50 |
| 70 | 50 | 50 |
| 75 | 50 | 75 |
| 80 | 50 | 75 |
| 85 | 75 | 75 |
| 90 | 75 | 75 |
| 95 | 75 | 100 |
| 99 | 100 | 100 |

Supplementary table 11 : Normative data for the SF-36 HC item expressed in centiles.

| Item | Reliability if dropped |
| --- | --- |
| sf01 | 0.87 |
| sf02 | 0.88 |
| sf03 | 0.88 |
| sf04 | 0.88 |
| sf05 | 0.88 |
| sf06 | 0.88 |
| sf07 | 0.88 |
| sf08 | 0.88 |
| sf09 | 0.88 |
| sf10 | 0.88 |
| sf11 | 0.88 |
| sf12 | 0.88 |
| sf13 | 0.88 |
| sf14 | 0.87 |
| sf15 | 0.88 |
| sf16 | 0.87 |
| sf17 | 0.87 |
| sf18 | 0.87 |
| sf19 | 0.87 |
| sf20 | 0.89 |
| sf21 | 0.89 |
| sf22 | 0.89 |
| sf23 | 0.87 |
| sf24 | 0.87 |
| sf25 | 0.87 |
| sf26 | 0.87 |
| sf27 | 0.87 |
| sf28 | 0.87 |
| sf29 | 0.87 |
| sf30 | 0.87 |
| sf31 | 0.87 |
| sf32 | 0.87 |
| sf33 | 0.88 |
| sf34 | 0.89 |
| sf35 | 0.88 |
| sf36 | 0.87 |

Supplementary Table 12 : Internal consistency and reliability of the MOS-SF36 if an item is dropped.

| Item | GH | MH | VT | BP | SF | RE | RP | PF |
| --- | --- | --- | --- | --- | --- | --- | --- | --- |
| S36 | 0.86 | - | - | - | - | - | - | - |
| S35 | 0.57 | - | - | - | - | - | - | - |
| S34 | 0.72 | - | - | - | - | - | - | - |
| S33 | 0.50 | - | - | - | - | - | - | - |
| S01 | 0.79 | - | - | - | - | - | - | - |
| S30 | - | 0.69 | - | - | - | - | - | - |
| S28 | - | 0.85 | - | - | - | - | - | - |
| S26 | - | 0.74 | - | - | - | - | - | - |
| S25 | - | 0.84 | - | - | - | - | - | - |
| S24 | - | 0.69 | - | - | - | - | - | - |
| S31 | - | - | 0.69 | - | - | - | - | - |
| S29 | - | - | 0.73 | - | - | - | - | - |
| S27 | - | - | 0.72 | - | - | - | - | - |
| S23 | - | - | 0.72 | - | - | - | - | - |
| S22 | - | - | - | 0.87 | - | - | - | - |
| S21 | - | - | - | 0.77 | - | - | - | - |
| S32 | - | - | - | - | 0.85 | - | - | - |
| S20 | - | - | - | - | 0.87 | - | - | - |
| S19 | - | - | - | - | - | 0.75 | - | - |
| S18 | - | - | - | - | - | 0.81 | - | - |
| S17 | - | - | - | - | - | 0.69 | - | - |
| S16 | - | - | - | - | - | - | 0.73 | - |
| S15 | - | - | - | - | - | - | 0.70 | - |
| S14 | - | - | - | - | - | - | 0.72 | - |
| S13 | - | - | - | - | - | - | 0.62 | - |
| S12 | - | - | - | - | - | - | - | 0.41 |
| S11 | - | - | - | - | - | - | - | 0.53 |
| S10 | - | - | - | - | - | - | - | 0.63 |
| S09 | - | - | - | - | - | - | - | 0.68 |
| S08 | - | - | - | - | - | - | - | 0.41 |
| S07 | - | - | - | - | - | - | - | 0.67 |
| S06 | - | - | - | - | - | - | - | 0.64 |
| S05 | - | - | - | - | - | - | - | 0.63 |
| S04 | - | - | - | - | - | - | - | 0.68 |
| S03 | - | - | - | - | - | - | - | 0.53 |

Supplementary Table 13 : Factor loadings for the MoS SF-36 Questionnaire
